# Treatment of Metabolic syndrome by combination of physical activity and diet needs an optimal protein intake: a randomized controlled trial

**DOI:** 10.1186/1475-2891-11-72

**Published:** 2012-09-17

**Authors:** Frédéric Dutheil, Gérard Lac, Daniel Courteix, Eric Doré, Robert Chapier, Laurence Roszyk, Vincent Sapin, Bruno Lesourd

**Affiliations:** 1 Clermont University, Blaise Pascal University, Laboratory of Metabolic Adaptations to Exercise in Physiological and Pathological conditions (AME2P, EA3533), BP 10448, F-63000, Clermont-Ferrand, France; 2 Sport Medicine and Functional Explorations, University Hospital (CHU) G. Montpied, F-63000, Clermont-Ferrand, France; 3 Occupational Medicine, Faculty of Medicine, F-63000, Clermont-Ferrand, France; 4 Geriatrics Departments, Faculty of Medicine, F-63000, Clermont-Ferrand, France; 5 Biochemistry, University Hospital (CHU) G. Montpied, F-63000, Clermont-Ferrand, France; 6 School of Exercise Science, Australian Catholic University, Locked Bag 4115 Fitzroy MDC VIC 3165, , Australia

**Keywords:** Protein intake, Physical activity, Metabolic syndrome, Albuminemia

## Abstract

**Background:**

The recommended dietary allowance (RDA) for protein intake has been set at 1.0-1.3 g/kg/day for senior. To date, no consensus exists on the lower threshold intake (LTI = RDA/1.3) for the protein intake (PI) needed in senior patients ongoing both combined caloric restriction and physical activity treatment for metabolic syndrome. Considering that age, caloric restriction and exercise are three increasing factors of protein need, this study was dedicated to determine the minimal PI in this situation, through the determination of albuminemia that is the blood marker of protein homeostasis.

**Methods:**

Twenty eight subjects (19 M, 9 F, 61.8 ± 6.5 years, BMI 33.4 ± 4.1 kg/m^2^) with metabolic syndrome completed a three-week residential programme (Day 0 to Day 21) controlled for nutrition (energy balance of −500 kcal/day) and physical activity (3.5 hours/day). Patients were randomly assigned in two groups: Normal-PI (NPI: 1.0 g/kg/day) and High-PI (HPI: 1.2 g/kg/day). Then, patients returned home and were followed for six months. Albuminemia was measured at D0, D21, D90 and D180.

**Results:**

At baseline, PI was spontaneously 1.0 g/kg/day for both groups. Albuminemia was 40.6 g/l for NPI and 40.8 g/l for HPI. A marginal protein under-nutrition appeared in NPI with a decreased albuminemia at D90 below 35 g/l (34.3 versus 41.5 g/l for HPI, p < 0.05), whereas albuminemia remained stable in HPI.

**Conclusion:**

During the treatment based on restricted diet and exercise in senior people with metabolic syndrome, the lower threshold intake for protein must be set at 1.2 g/kg/day to maintain blood protein homeostasis.

## Introduction

The International Diabetes Federation (IDF) defines the metabolic syndrome (MS) as the co-occurrence of any three of the five following abnormalities : abdominal obesity (waist circumference > 94 cm in men and > 80 in women), dyslipidemia (triglyceridemia > 1.5 mmol/l, HDL cholesterol < 0.4 g/l in men and < 0.5 g/l in women), blood pressure (BP) > 130/85 and/or medical treatment, and fasting glycemia > 5.55 mmol/l and/or medical treatment [1]. MS is associated with an increased risk of cardiovascular diseases [2] and prevalence of type 2 diabetes [3]. In developed countries, its increasing prevalence is mainly linked to obesity and age [4].

The most efficient strategy to counteract MS is a significant reduction in caloric intake associated with an increase in physical activity (PA). Such programmes aim primarily to reduce overweight, the most visible manifestation of MS, but the challenge is to reduce the fat mass without affecting lean body mass, especially in senior, for whom a progressive loss of muscle mass and strength is a natural phenomenon [5], even in those who are healthy and physically active [6]. In addition, the recovery of skeletal muscle mass in ageing people is impaired after a catabolic state [7,8]. Physical exercise and an adequate protein intake are of prime importance in preventing muscle loss. However, there is no consensus on the adequate level of protein intake in the case of senior patients undergoing a combined treatment of caloric restriction and physical activity (PA) for MS. In these patients, age, exercise and energy restriction increase protein requirement.

The recommended dietary protein allowance (RDA) for the general population has been set at 0.8 g/kg/day [9,10]. RDA is defined as the average daily dietary intake level that is sufficient to meet the nutrient requirements of nearly all healthy individuals. RDA corresponds to the mean lower threshold intake (LTI) of a panel of healthy people plus two standard deviations, including 97.5% of the population, and is calculated as 1.3 LTI day [9].

Some guidelines recommend increasing RDA to 1.0–1.3 g/kg/day in senior [11].

PA increases the need for proteins whatever the age of the subject [9,12,13], and this specificity must be taken into account in senior people [14,15].

Total energy intake has a protein sparing effect [16-18]. Conversely insufficient energy intake will increase the protein needed to compensate for the energy deficit. As skeletal muscle is the main storage site of body proteins and amino acids, this will lead to an undesirable reduction of muscle mass [19]. Excessive protein intake is of no value, in particular because it will over-exert the kidney [20] and increase the end products of protein metabolism (urea and uric acid). It will also increase the intake of undesirable saturated fatty acids via proteins of animal origin [21]. The precautionary principle is to bear this factor in mind in senior patients, since age-related renal insufficiency is common [22], especially in people with elevated BP [23] and/or dyslipidemia [24], which is often the case in subjects with MS.

The challenge for the prescriber is to give neither too much nor too little protein, in order to preserve the muscle mass without inducing harmful effects on the kidney in older subjects with MS.

Our aim in the present study was to assess the minimal need for proteins in a population of senior MS subjects. There are a limited number of tools to assess the appropriate level of protein intake. One way is to control preservation of muscle mass over a long period, but this can only be done in animal studies for ethical reasons. Nitrogen balance studies are probably the gold standard, but they are rather cumbersome to perform. Monitoring the levels of albumin, the blood marker of protein metabolism homeostasis, seems to be the most convenient index and was chosen for this study. Moreover, albumin levels are closely linked to morbidity, and represent a large consensus to assess nutritional status [25,26]. We decided to determine protein LTI by recording albumin levels in older subjects with MS participating in a weight reduction programme including exercise and energy restriction. The programme comprised two parts: a three-week residential programme during which subjects stayed in a medical establishment on a controlled diet with regular PA, and a six-month follow-up at home.

## Subjects and methods

### Participants

We needed to recruit between 25 and 30 volunteers, of both sexes, aged from 50 to 70 years, presenting the characteristics of the MS as defined by the IDF criteria in 2005 [1]. Potential participants underwent a comprehensive medical screening procedure. Volunteers were eligible for inclusion in the study if they had a sedentary lifestyle, and stable body weight over the previous year (i.e., had not fluctuated more than 2 kg), and if their medical treatment had remained the same during the 6 months before recruitment. Major criteria for exclusion were the presence of cardiovascular, hepatic, renal or endocrine diseases, the use of medications that affect body weight, restricted diet in the past year, insufficient motivation as assessed by interview, and inability to complete a maximal exercise tolerance test (VO2max).

**Figure 1:**
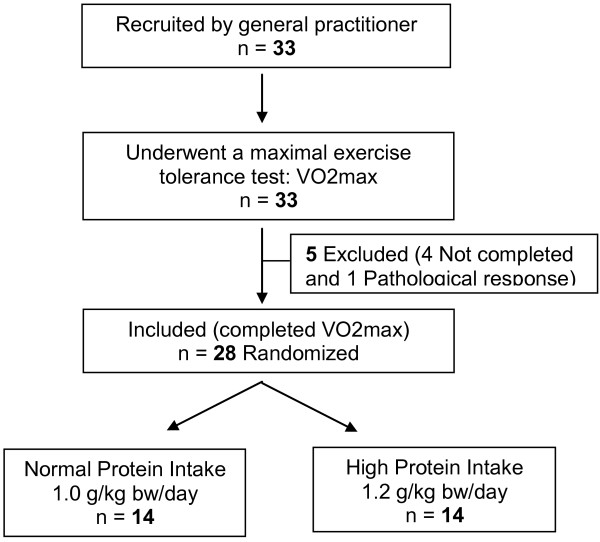
Flow chart of the study design.

Of the 33 participants with MS recruited by their general practitioner, 4 were unable to complete a maximal exercise tolerance test (VO2max) and 1 had a pathological response. Twenty eight volunteers, 19 men, 9 women, aged 61.8 ± 6.5 years, were included. All were Caucasians. They were randomly assigned to two groups of different PI, normal and high. They all completed the study (Figure 1).

The study was approved by the local “Committee for the Protection of the Person for Research in Biology” (CPPRB). All participants gave written informed consent. They were informed that the study would be comparing diets with two different protein intakes and that they would be assigned a diet at random. Random assignments to one of two different protein intake groups were computer-generated after subjects were considered eligible to take part.

### Study outcomes

The primary outcome was the change from baseline in albumin levels. Secondary outcomes included other markers of protein intake such as body composition, in particular lean mass, creatinine levels, renal clearance and pro-inflammatory factors such as C-Reactive Protein (CRP) and orosomucoid.

### Study design

In this 26-week study, participants were randomly assigned, with stratification according to sex and weight, to one of two groups: a normal PI group (NPI) with intake of 1.0 g/kg/d and a high PI group (HPI) with 1.2 g/kg/d.

**Figure 2:**
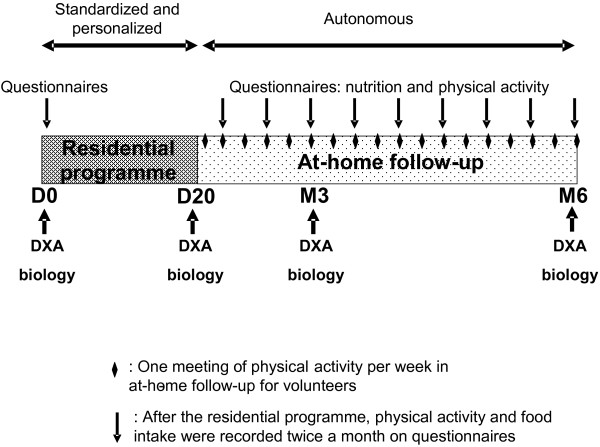
**Study design:** The two groups of volunteers (Normal and High protein-intake) followed a three-week residential programme with standardized and personalized diet and physical activity. Thereafter they returned home and were autonomous to manage their diet and physical activity, the latter being accompanied by a weekly session of physical activity on a voluntary basis. The two groups differed only by protein intake.

The study design is shown in Figure 2. The study comprised three chronological stages: Day 0 (D0), a 3-week residential programme (Day 0 to Day 21) and at-home follow up (D20 to D180). Clinical, biological and body composition parameters were measured at D0, D20, D90 and D180.

#### At D0

Basal metabolic rate (BMR) was calculated by the equations of Black [27]: BMR = 0.963 . weight^0.48^ . height^0.50^ . age^-0.13^ for women, BMR = 1.083 . weight^0.48^ . height^0.50^ . age^-0.13^ for men. Anthropometrical and clinical values (weight, size height, BMI, waist circumference, blood pressure), body composition and biological parameters were measured. Before commencing the study, patients completed questionnaires concerning their food intake and PA over the previous week. Daily energy intake (DEI) and daily energy expenditure (DEE) were estimated from the self reported questionnaires. The food intake questionnaires identified possible deficiencies.

#### During the three-week residential programme

The subjects carried out daily individually adapted physical activities with a coach: walking (2 hours), aquagym (1 h, 3 times/week), keep fit activity (1 h, 3 times/week). Exercise intensity was fixed between 40 and 60% of the heart rate reserve (maximum theoretical heart rate – resting heart rate), by a heart rate recorder (Polar 4000). They followed the same programme between both groups six days a week. On the 7^th^ day they only walked. Daily throughout the residential program, the patients received both standard and personalized balanced meals drawn up by dieticians. Their total daily food intake was calculated in order to reach a negative energy balance (EB = DEE - DEI) of 500 kcal/day. They also attended lectures on the MS, nutrition physiology, cooking and physical-activity. The aim of this educational support was to make them aware of the lifestyle they would need to adopt in order to maintain the beneficial effects of the regimen followed.

#### From D20 to D180

The subjects returned home and were left in charge of managing the programme by themselves. They were asked to carry out the same training program and the same diet. Thereafter, PA sessions were organized once a week (keep fit activity or aquagym) to maintain compliance with the programme. They completed a questionnaire twice a month on their PA and eating habits. A dietician and a physical coach could be contacted if they had any queries.

### Methods

Clinical follow up was performed by a physician and psychological follow-up to verify the treatment acceptance was assessed by a psychologist. Daily energy intake (DEI) and physical activity index were based on questionnaires before and after the residential programme (three-day food intake and PA recorded once every 15 days). DEE was quantified by recording the time and intensity of each PA and the physical activity index (PAI = DEE/BMR) was calculated [28]. Each day during the residential programme, the patients received both standard and personalized balanced meals drawn up by dieticians. The aim was to restore macronutrient balance: 30 to 35% lipids, 15 to 20% proteins and carbohydrates for the rest. The PI difference of 0.2 g/kg/d between NPI and HPI represented, for a mean weight of 80 kg, about 16 g/d of protein per participant, a difference of 64 kcal/day. This difference was compensated for by the addition of the same quantity of carbohydrates for the NPI group. In both groups, daily PA was programmed for each subject to obtain a PAI equal to 1.4 [28,29].

### Biometry and anthropometry

All subjects underwent medical examinations. Body height was measured with a stadiometer, BMI was calculated as the weight in kilograms divided by the square of the height in meters. Waist circumference and BP were recorded. Body composition was assessed by dual energy X-ray absorptiometry (DXA) (Hologic QDR Delphi series; Waltham, USA). The in vivo coefficients of variation were 4.2 and 0.48% for fat and lean mass, respectively. Central fat, (as a surrogate for visceral fat), was assessed by DXA, which measured the % fat in a rectangle from the upper edge of the second lumbar vertebra to the lower edge of the fourth lumbar vertebra. The vertical sides of this area were the continuation of the lateral sides of the rib cage [30]. All measurements for a given parameter were made by the same investigator.

### Biochemical measurements

Fasting blood samples were taken between 6.30 and 7.30 a.m., aliquoted and stored at −80°C until analyses. Basic biological examinations (glucose, lipid, creatinine, CRP and orosomucoid levels) were performed in the biochemistry laboratory of the University Hospital. Renal clearance was assessed by Cockcroft’s formula [31].

### Statistical analysis

Gaussian distribution of the data was tested by the Kolmogorov-Smirnov test. Data are presented as means ± standard error (SE). Significance was accepted at the p < 0.05 level. Statistical procedures were performed by SPSS Advanced Statistics software version 17 (SPSS Inc., Chicago, IL).

Using the method described by Howell [32], we calculated the number of subjects needed for a significant change in albumin levels between groups (based upon preliminary data). The minimum number was 10 participants per group for a p < 0.05 and a type II error of 10%. Under these conditions the statistical power was 90%.

Baseline characteristics were compared by analysis of variance or Fisher’s exact test. Longitudinal changes between groups were tested with the use of mixed-model repeated-measures analysis of variance, with adjustment for baseline values and sex. The primary focus of the analyses was the 3-month change in albumin levels in the two groups.

In the event of interaction between the repeated measurements (time effect) and the main factor (group of protein intake), the changes within a group were analyzed either by a Newman-Keuls post-hoc test for normally distributed data, or a nonparametric Wilcoxon test for non-normally distributed data.

Relationships between energy balance, physical activity and other data were assessed either by Pearson correlation or by multiple regression analysis.

## Results

### Descriptive characteristic of participants at baseline

The descriptive characteristics of volunteers before the residential programme are presented in Table 1. There was no difference at baseline between groups. Usual food intake as indicated on questionnaires revealed that all patients of both groups had high lipid and low carbohydrate consumption, with a high ratio of high/low glycemic index (Table 1). Mean cholesterol consumption was 341 ± 97 mg/d. The mean DEE before the residential programme was 1983 ± 228 kcal/day corresponding to a low PAI of 1.22 ± 0.09.

**Table 1:** Baseline characteristics of participants*

**Characteristic**	**Normal protein intake (n = 14)**	**High protein intake (n = 14)**	**Significance**
Age – years	62.9 ± 6.9	60.6 ± 6.0	NS
Male (M) : n (%)	10 (71)	9 (64)	NS
Female (F) : n (%)	4 (29)	5 (36)	NS
Weight (kg) – M	90.4 ± 8.7	96.3 ± 4.1	NS
Weight (kg) - F	90.0 ± 18.7	88.7 ± 14.5	NS
BMI (kg/m^2^)	32.1 ± 4.2	35.2 ± 4.2	NS
MS parameters			
BMI (kg/m^2^)	32.1 ± 4.2	35.2 ± 4.2	NS
Waist circumference (cm) – M	106.5 ± 7.7	104.3 ± 18.5	NS
Waist circumference (cm) – F	101.5 ± 5.8	100.6 ± 9.2	NS
Blood Pressure (mmHg)	137/83 ± 11/5	135/86 ± 18/13	NS
Triglycerides (mmol/l)	1.68 ± 1.15	1.88 ± 0.55	NS
HDL (mmol/l)	1.44 ± 0.42	1.06 ± 0.33	NS
Glycemia (mmol/l)	6.15 ± 1.86	5.13 ± 0.68	NS
Use of medication: n (%)			
Antihypertensive agents	5 (36)	6 (29)	NS
Lipid lowering drugs	3 (21)	4 (29)	NS
Hypoglycemiant drugs	3 (21)	5(36)	NS
Basal Metabolic Rate	1626 ± 178	1587 ± 254	NS
Daily Energy Expenditure (kcal/d)	1983 ± 229	1920 ± 307	NS
Physical Activity Index	1.22 ± 0.09	1.19 ± 0.12	NS
Daily Energy Intake (kcal/d)	2073 ± 556	1921 ± 348	NS
Daily Energy Intake (kcal/kg/d)	23.8 ± 6.5	21.2 ± 4.9	NS
Percentage of each macronutrient in the food			
% Carbohydrates	39.2 ± 5.5	40.5 ± 6.0	NS
of high glycemic index carbohydrates	13.9 ± 5.3	13.0 ± 1.2	NS
% Lipids	44.5 ± 4.7	41.9 ± 4.4	NS
% Proteins	16.3 ± 1.5	17.6 ± 2.7	NS
Protein intake (g/kg/d)	0.91 ± 0.26	0.90 ± 0.22	NS

*: Plus–minus values are means ± SD.

### Descriptive characteristics during the residential programme

#### Intervention

Food intake over the six months is presented in Table 2, and energy balance and PAI in Table 3. DEI decreased and DEE increased during the residential programme, both significantly, resulting in a negative balance. The PAI was set at 1.4 ± 0.1. Macronutrient distribution improved: lipid intake was lower and that of carbohydrates and proteins higher. There were reduced amounts of saturated fatty acids and cholesterol (Table 2).

**Table 2:** Changes in food intakes: percentage of each macronutrient (carbohydrates, lipids, proteins)

**Variable**	**Groups**	**D0**	**D20**	**D90**	**D180**
% Carbohydrates	NPI	39.2 ± 5.5	48.3 ± 2.5**†**	42.5 ± 4.6**†**	39.1 ± 3.4
	HPI	40.5 ± 6.0	47.3 ± 3.2**†**	41,6 ± 4.6	39.5 ± 4.0
% Lipids	NPI	44.5 ± 4.7	32.8 ± 2.1**†**	38.2 ± 4.7	41.7 ± 3.6
	HPI	41.9 ± 4.4	28.7 ± 3.4**†**	35.5 ± 5.2	38.1 ± 5.6
% Proteins	NPI	16.3 ± 1.5	18.9 ± 0.8*	19.4 ± 0.19†*	19.4 ± 1.3†*
	HPI	17.6 ± 2.7	24.8 ± 1.6†*	23.0 ± 2.3†*	22.2 ± 2.2†*
Protein Intake (g/kg/d)	NPI	0.91 ± 0.26	0.95 ± 0.11*	0.94 ± 0.19*	0.96 ± 0.17*
	HPI	0.90 ± 0.22	1.19 ± 0.13†*	1.10 ± 0.14†‡*	1.09 ± 0.20†‡*

All calculations were done on Bilnut program using CIQUAL (S.C.D.A. Nutrisoft, Le Hallier 37390 Cerelles, France).

* : p < 0.05 to compare the percentage change between D0 and D20, D90 and D180 in the two groups (High Protein Intake versus Normal Protein Intake) using the Bonferroni test.

**†** : p < 0.05 to compare the value at the follow-up time with the baseline value (D0) within each group, as calculated by mixed-model repeated-measures analysis of variance.

**‡** : p < 0.05 to compare the value at the follow-up time with the end of the residential programme (D20) within each group, as calculated by mixed-model repeated-measures analysis of variance.

**Table 3:** Changes in energy balance, physical activity index, MS parameters and body composition during the residential programme and follow-up for the two groups of protein intake (PI): Normal (NPI) and High (HPI) with respectively a PI at 1.0 g/kg/day and 1.2 g/kg/day

**Variable**	**Groups**	**D0**	**D20**	**D90**	**D180**
Body composition measured by DEXA:					
Weight (kg)	NPI	90.3 ± 11.6	86.5 ± 11.0**†**	84.4 ± 11.1**†‡**	85.4 ± 12.1**†‡**
	HPI	94.1 ± 15.2	90.7 ± 14.5**†**	87.1 ± 13.6**†‡**	86.4 ± 15.0**†‡**
BMI (kg/m^2^)	NPI	32.1 ± 4.2	30.7 ± 3.8**†**	29.9 ± 3.5**†‡**	30.3 ± 3.7**†‡**
	HPI	35.2 ± 4.2	33.9 ± 4.1**†**	32.6 ± 4.3**†‡**	32.4 ± 4.7**†‡**
Lean (kg)	NPI	58.287 ± 7.47	57.323 ± 7.56**†**	56.488 ± 7.89**†**	56.798 ± 7.99**†**
	HPI	56.594 ± 11.04	55.987 ± 10.32	55.223 ± 10.57**†**	55.137 ± 10.47**†**
Total fat (kg)	NPI	29.542 ± 9.75	26.737 ± 9.38**†**	25.524 ± 8.94**†**	26.213 ± 9.580**†**
	HPI	35.229 ± 8.25	32.474 ± 7.72**†**	29.598 ± 7.68**†‡**	29.068 ± 9.18**†‡**
Visceral fat (kg)	NPI	3.277 ± 1.24	2.839 ± 1.22**†**	2.695 ± 1.07**†**	2.527 ± 1.01**†‡**
	HPI	3.295 ± 0.88	2.916 ± 0.79**†**	2.662 ± 0.75**†**	2.445 ± 0.71**†‡**
Fat percentage (%)	NPI	32.3 ± 7.9	30.5 ± 8.3**†**	29.9 ± 8.2**†**	30.2 ± 8.3**†**
	HPI	37.4 ± 6.1	35.7 ± 5.9**†**	34.0 ± 6.6**†**	33.4 ± 7.2**†**
Energy Balance (kcal/d)	NPI	+ 92 ± 521	−751 ± 147**†**	−521 ± 304**†‡**	−413 ± 304**‡**
	HPI	+ 34 ± 348	−635 ± 102**†**	−524 ± 83**†‡**	−444 ± 71**‡**
Physical Activity	NPI	1.22 ± 0.09	1.42 ± 0.07**†**	1.31 ± 1.97**†‡**	1.30 ± 0.07**†‡**
	HPI	1.19 ± 0.12	1.40 ± 0.09**†**	1.33 ± 1.82**†‡**	1.28 ± 0.04**†‡**
Metabolic Syndrome parameters:					
Waist circumference (cm)	NPI	105.1 ± 7.4	102.7 ± 7.9**†‡**	99.2 ± 6.3**†‡**	98.9 ± 7.4**†‡**
	HPI	101.6 ± 11.8	97.2 ± 9.6**†‡**	95.1 ± 9.9**†‡**	93.3 ± 9.1**†‡**
Blood Pressure (mmHg)	NPI	137/83 ± 11/5	130**†**/80 ± 13/5	129**†**/80 ± 15/6	132/78 ± 16/9
	HPI	135/86 ± 19/13	128**†**/80 ± 15/16	125**†**/85 ± 16/18	127/82 ± 20/16
Triglycerides (mmol/l)	NPI	1.68 ± 1.15	1.19 ± 0.34	1.17 ± 0.40	1.27 ± 0.61
	HPI	1.88 ± 0.55	1.37 ± 0.26	1.85 ± 0.21	1.86 ± 1.07
HDL (mmol/l)	NPI	1.44 ± 0.42	1.49 ± 0.31	1.31 ± 0.31**‡**	1.58 ± 0.46
	HPI	1.06 ± 0.33	1.02 ± 0.29	1.13 ± 0.33	1.14 ± 0.25
Glycemia (mmol/l)	NPI	6.15 ± 1.86	6.26 ± 1.76	5.7 ± 1.97**‡**	6.28 ± 2.34
	HPI	5.13 ± 0.68	4.54 ± 0.54**†**	4.98 ± 0.79	4.92 ± 0.50
Other Lipid parameters:					
Total cholesterol (mmol/l)	NPI	6.08 ± 1.46	5.07 ± 0.95**†**	5.13 ± 1.25**†**	5.56 ± 0.89
	HPI	5.79 ± 1.07	4.65 ± 1.09**†**	6.18 ± 1.60	6.19 ± 1.08
LDL (mmol/l)	NPI	3.79 ± 1.21	3.05 ±0.87**†**	3.47 ± 1.25	3.43 ± 0.95
	HPI	3.89 ± 1.01	2.99 ±0.99**†**	4.21 ± 1.34	4.25 ± 0.97
Albumin levels (g/l)	NPI	40.6 ± 3.3	40.3 ± 3.1	34.3 ± 1.8†‡*	35.3 ± 2.2**†‡**
	HPI	40.8 ± 2.4	41.3 ± 2.6	41.5 ± 2.6*	41.0 ± 3.2*
Pro-inflammatory factors:					
CRP (mg/l)	NPI	5.09 ± 4.06	3.64 ± 3.69	4.18 ± 5.60	3.68 ± 4.12
	HPI	4.19 ± 2.33	3.80 ± 3.48	3.04 ± 3.23	2.99 ± 2.05
orosomucoid (mg/l)	NPI	0.85 ± 0.16	0.80 ± 0.19	0.75 ± 0.19	0.84 ± 0.15
	HPI	0.91 ± 0.29	0.80 ± 0.33	0.91 ± 0.25	0.84 ± 0.41
Renal function:					
creatinine levels (mmol/l)	NPI	89.4 ± 19.9	89.1 ± 18.8	80.1 ± 20.7	81.1 ± 20.4
	HPI	84.9 ± 22.2	88.2 ± 24.1	86.1 ± 23.7	84.0 ± 21.3
Cockroft (ml/min)	NPI	100.3 ± 28.3	95.4 ± 24.2	107.1 ± 27.9	106.6 ± 32.0
	HPI	111.8 ± 23.7	104.5 ± 24.9	106.7 ± 23.4	109.0 ± 24.4

* : p < 0.05 to compare the percentage change between D0 and D20, D90 and D180 in the two groups (High Protein Intake versus Normal Protein Intake) using the Bonferroni test.

**†** : p < 0.05 to compare the value at the follow-up time with the baseline value (D0) within each group, as calculated by mixed-model repeated-measures analysis of variance.

**‡** : p < 0.05 to compare the value at the follow-up time with the end of the residential programme (D20) within each group, as calculated by mixed-model repeated-measures analysis of variance.

#### General effects for both groups

At the end of the residential programme, the combination of diet and PA had significantly reduced body weight, BMI, waist circumference and systolic BP. Fifty seven percent of weight loss was in fat mass, with a significant decrease in central fat. The rates of HDL remained stable and triglyceride levels had an overall tendency to decrease (p < 0.1). Other blood lipid parameters decreased significantly at D20 and then returned to baseline levels. Creatinine levels and creatinine clearance, as assessed by Cockroft’s formula remained stable (Table 3).

Following the residential programme, dietary recommendations were given every 15 days to maintain a negative energy balance. The patients gradually went back to their former eating habits with increased lipid intake and a reduction in carbohydrates. Weight loss and central fat loss continued, as did the reduction in BMI and waist circumference, with no significant difference between groups (Table 3).

### Main judgment criteria

At D20, there was no significant change in albumin levels in either group. Following the residential programme, the levels decreased in NPI group at D90 and D180, to reach the threshold value of 35 g/l considered as a marker of a protein deficiency. Albumin levels in the HPI group remained stable throughout the experimental period.

### Secondary judgment criteria

In both groups, creatinine levels and creatinine clearance remained stable at all times, as did CRP and orosomucoid.

## Discussion

**Figure 3:**
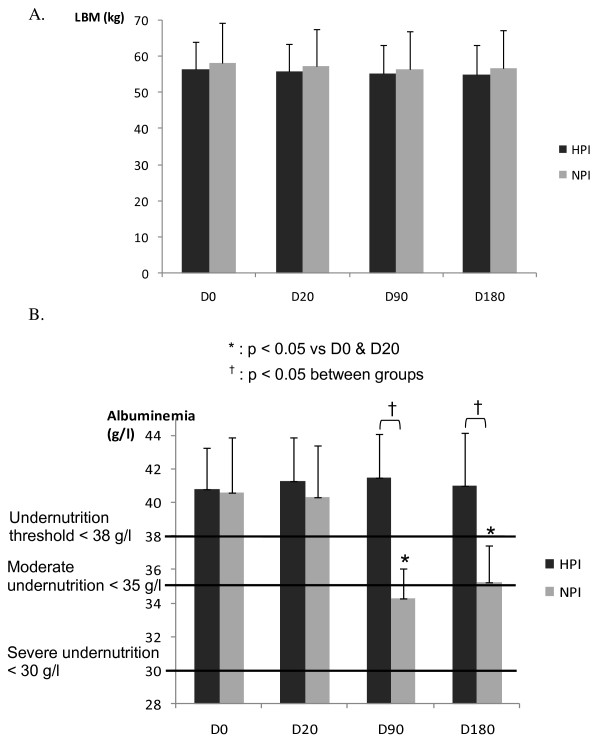
**Change from baseline (day 0) to Month 6 (day 180) in albumin levels and lean body mass (LBM) for both groups: normal protein intake (NPI) set at 1.0 g/kg/d and high protein intake (HPI) at 1.2 g/kg/d.** Solid bars represent albumin levels or lean mass in High protein intake group. Open bars represent albumin levels or lean mass in Normal protein intake group. T bars indicate standard errors. Panels **A** and **B** show the change in lean mass and albumin levels, respectively, for all participants (n= 28), who were randomly assigned to a High protein intake (n= 14) or to a Normal protein intake (n= 14). No missing data.

Our study shows that when PA and nutritional habits are modified by a healthier lifestyle, there is a significant improvement in MS criteria (Table 3). When subjects entered the study, protein intake was about 0.9 g/kg/d, the level currently considered as adequate for the senior [10] and albumin levels were normal (Table 3, Figure 3). As the exercise regimen and the reduced caloric intake of the programme were expected to increase the protein LTI, we set protein intake at 1 g/kg/d for NPI and at 1.2 g/kg/d for HPI.

We measured albumin levels at the beginning of the study (D0) and at D20, D90 and D180 in the two groups of MS patients older than 50 years. There was no change in the levels in the HPI group, while in the NPI group, they were lower at D90. This significant decrease would probably indicate a too low protein intake in the NPI group, given the new conditions of PA and overall caloric intake [9,12-14]. In contrast, the PI set at 1.2 g/kg/d kept albumin levels steady. The fall in level was observed at D90 only and not at D20, probably due to its long half-life of 20 days.

A catabolic phase may occur in the event of increased inflammatory status [33]. In the present study, we monitored this status by assaying CRP, which remained stable with normal levels in both groups (Table 3). Likewise, altered renal function may be the cause of hypoalbuminemia. This was not the case in our patients, as assessed by normal and stable renal Cockcroft clearance (Table 3). Moreover, high protein intake is now considered to be only a weak risk for renal function in healthy senior individuals [34]. Finally, all patients were free of medications which may influence albumin levels.

Consequently, our study shows that LTI (and not RDA) for protein must be set at 1.2 g/kg/d when physical activity together with reduced caloric intake are prescribed to patients suffering from MS. This result agrees with the findings of Lucas & Heiss [15], who proposed a RDA of 1–1.3 g/kg/d for older adults (> 50 years old) engaged in physical training corresponding to a LTI of 0.8 to 1.0 g/kg/day. The addition of caloric restriction, as in this study, increases LTI for protein to 1.2 g/kg/d.

Such a level of protein intake may appear high for people consuming light meals. For an individual weighing 80 kg, it represents 100 g/day of protein dry weight, that is to say 500 g of crude protein. Dietary animal protein is the primary source of high biological value protein [35]. If fifty percent of protein intake is from animal origin, this corresponds to 250 g meat or fish per day, since eggs and cheese are drastically reduced or even suppressed on account of their high lipid content. We may also consider that 100 g protein represent 400 kcal. If the total caloric intake is set at 2000 kcal, proteins will represent 20% of intake.

## Conclusion

This study is a contribution to the quantification of the optimal protein lower threshold intake for individuals suffering from metabolic syndrome entering a programme of weight reduction with controlled diet and exercise. This is an important issue because insufficient protein intake could be detrimental when physical activity is added to a restricted diet. Our study suggests protein intake should be 1.2 g/kg/d in senior people suffering from metabolic syndrome and taking part in a weight loss programme.

## Competing interests

The authors declare that they have no competing interests.

## Authors’ contributions

FD has participated as PhD student and main investigator. BL, DC and GL contributed to the conception of the protocol, data analysis and manuscript drafting. LR and VS measured all biologic data. RC had responsibilities in daily diet. ED managed physical activity. GL has revised and given final approval of the manuscript. All authors have read and approved the final manuscript.
